# Leakage and Stenosis of the Hepaticojejunostomy Following Surgery for Perihilar Cholangiocarcinoma ^**†**^

**DOI:** 10.3390/jcm9051392

**Published:** 2020-05-08

**Authors:** Jan Bednarsch, Zoltan Czigany, Daniel Heise, Sven Arke Lang, Steven W. M. Olde Damink, Tom Luedde, Philipp Bruners, Tom Florian Ulmer, Ulf Peter Neumann

**Affiliations:** 1 Department of Surgery and Transplantation, University Hospital RWTH Aachen, 52074 Aachen, Germany; jbednarsch@ukaachen.de (J.B.); zczigany@ukaachen.de (Z.C.); dheise@ukaachen.de (D.H.); svlang@ukaachen.de (S.A.L.); steven.oldedamink@maastrichtuniversity.nl (S.W.M.O.D.); fulmer@ukaachen.de (T.F.U.); 2 Department of Surgery, Maastricht University Medical Centre (MUMC), 6229 Maastricht, The Netherlands; 3 Department of Medicine III, University Hospital RWTH Aachen, 52074 Aachen, Germany; tluedde@ukaachen.de; 4 Department of Radiology, University Hospital RWTH Aachen, 52074 Aachen, Germany; pbruners@ukaachen.de

**Keywords:** hepaticojejunostomy, anastomotic leakage, anastomotic stenosis, perihilar cholangiocarcinoma (pCCA)

## Abstract

This study aims to provide a deep insight into the incidence and clinical significance of postoperative anastomotic leakage (AL) and anastomotic stenosis (AS) of the hepaticojejunostomy (HJ) after curative-intent liver resection for perihilar cholangiocarcinoma (pCCA). Between 2011 and mid-2019, 114 patients with pCCA underwent surgery in curative intent at our institution and were analyzed regarding the postoperative incidence of AL and AS. Further, associations between AL and AS and clinical characteristics were assessed using multiple univariate logistic regression analyses. AL was diagnosed in 11.4% (13/114) of the patients resulting in postoperative mortality in the minority of patients (23.0%, 3/13). AS occurred in 11.0% (11/100) of the individuals eligible for follow-up with local tumor recurrence being the underlying pathology in 72.7% (8/11) of the cases. None of the investigated clinical factors including surgical difficulty of the HJ showed a meaningful association with AL or AS. AL and AS are frequent complications and can be treated by conservative, interventional or surgical therapy with a high success rate. Also, technical difficulty of the HJ appears not to be not associated with the occurrence of AL or AS. Moreover, AS is associated with tumor recurrence in the majority of cases.

## 1. Introduction

Cholangiocellular carcinoma (CCA) is the second most common malignancy of the liver harboring a dismal oncologic prognosis as CCAs are generically diagnosed at advanced disease stages [1,2,3]. Surgical resection has evolved as the mainstay of treatment for patients with resectable CCA, since the oncological outcome with reported five-year survival rates to up to 60% in selected cohorts is superior to interventional or systemic therapies [4,5]. However, surgical resection for CCA is usually associated with a significant perioperative mortality up to 12% in contrast to partial hepatectomies for other malignancies which are generally considered safe in experienced high-volume centers [6,7,8]. 

Perioperative outcome is in particular impaired in the most frequent subtype perihilar CCA (pCCA). As the tumor is per definition located at the liver hilum, major hepatectomies with vascular resection are essentially required to achieve a complete tumor removal [4,9,10]. The resection of the extrahepatic and intrahepatic biliary tree leaves us with multiple, often small segmental bile ducts at the resection plane of the remnant liver which have to be reconstructed. These complex hepticojejunostomies (HJs) are burdened with technical challenges and possible pitfalls for the perioperative outcome in these patients.

Albeit the difficulty of the HJ is well-known among hepatobiliary surgeons, no comprehensive data are available on the incidence and clinical significance of anastomotic leakage (AL) and anastomotic stenosis (AS) following HJ in pCCA patients. AL is mainly investigated after pancreaticoduodenectomy and reported to occur in 2% to 8% of all patients undergoing the procedure [11,12,13]. Risk factors for postoperative leakage in this scenario are an increased body mass index (BMI), preoperative endoscopic biliary drainage (EBD) and an anastomosis on segmental bile ducts [14]. However, these findings are hardly transferable to HJs after surgery for pCCA. The same scarcity of data applies for the available evidence in terms of AS following partial hepatectomies and HJ for pCCA. Biliary stenosis is the most common long-term complication after HJ, which is reported to occur in 3% to 13% in various surgical scenarios and disease entities [15,16,17].

Thus, we aimed to investigate the incidence, clinical consequences and risk factors for AL and AS in a large monocentric cohort of pCCA patients undergoing surgical resection.

## 2. Materials and Methods

### 2.1. Patients

One hundred fifteen (*n* = 115) consecutive patients with localized pCCA and no signs of systemic disease underwent first-line surgical resection at the University Hospital RWTH Aachen (UH-RWTH) between January 2011 and June 2019. One of these patients underwent resection of the extrahepatic biliary tree without concomitant partial hepatectomy and was therefore excluded from the analysis (*n* = 114). The study was conducted at the UH-RWTH in accordance with the requirements of the Institutional Review Board of the RWTH-Aachen University (EK 430/19), the current version of the Declaration of Helsinki, and the good clinical practice guidelines (ICH-GCP). Demographic, clinical, and histo-pathological characteristics are shown in Table 1.

### 2.2. Staging and General Surgical Technique

All patients who were referred for surgical treatment to our institution underwent a detailed clinical work-up as previously described [18]. The indication for surgery as the primary treatment and the selection of the particular operative procedure was made by an experienced hepatobiliary surgeon. Preoperative endoscopic biliary drainage (EBD) or percutaneous biliary drainage (PBD) were carried out in patients presenting with clinically relevant biliary obstruction with EBD being generally preferred over PBD. We advocate a unilateral stenting strategy to relieve the future liver remnant (FLR) from cholestasis. Bilateral stenting was performed on occasions where complications of the contralateral biliary system occurred, such as persisting cholangitis. In individuals with insufficient FLR scheduled for right-sided hepatectomy, a right portal vein embolization (PVE) was performed 2 to 4 weeks before surgery. For this purpose, the standardized FLR (sFLR) was calculated for every patient [19]. The surgical resection was performed, as previously described, either as a right- or left sided hepatectomy (hilar en-bloc resection) in a no-touch technique with mandatory portal vein reconstruction [4,18].

**Figure 1:**
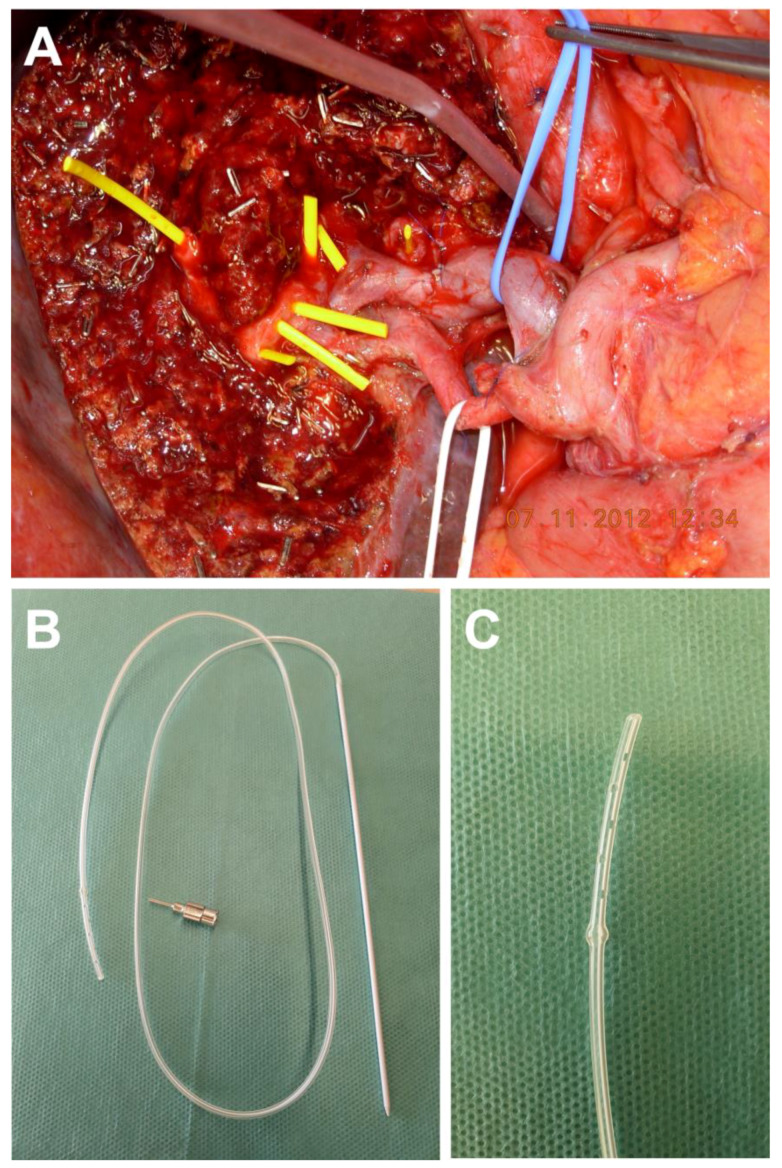
Intraoperative view after extended left hepatectomy for perihilar cholangiocarcinoma. (**A**) Multiple bile ducts are located in the resection plane (bile ducts are probed with yellow plastic tubes for demonstration purposes). (**B**,**C**) The trans-anastomotic internal-external drainages used in the cohort of this paper is shown (PancreaPlus, Peter Pflugbeil GmbH, Zorneding, Germany).

The applied surgical technique for the HJ comprised an open hand-sewn anastomosis technique with an end-to-side HJ followed by a Roux-en-Y reconstruction with an end-to-side jejunojejunostomy in a retrocolic fashion. After liver resection, hemostasis was achieved and all separate bile ducts in the resection plane were identified. Bile ducts in close proximity were approximated to form a common ostium by interrupted sutures (PDS 5-0; Ethicon, Hamburg, Germany). If approximation of the separate bile ducts was technically not feasible, separate HJs were carried out. The posterior wall of the HJ was created using interrupted sutures (PDS 5-0). Prior to creation of the anterior wall of the anastomosis in a similar fashion, one or more trans-anastomotic internal-external drainages (PancreaPlus, Peter Pflugbeil GmbH, Zorneding, Germany) were placed in the bile ducts and secured with an absorbable suture (PDS 5-0) (Figure 1). While the distal end of the drainage was positioned in the intrahepatic bile duct, the proximal end was pierced through the jejunal wall of the hepatobiliary limb of the Roux-en-Y reconstruction. After creating a Witzel’s channel around the drain, it was finally led through the abdominal wall and secured at the level of the skin with a non-absorbable suture. The number of the drains used and decision which particular bile ducts required drainage were made on a case-by-case basis and surgeon’s preference. After completion of the HJ, a white test using each internal-external drainage was performed as intraoperative bile leakage test [20]. The internal–external drainages were left open postoperatively to observe the bile production of the remnant liver. In all cases, cholangiography was performed on the fifth postoperative day. If no bile leakage was detected, the drains were subsequently closed but remained in situ. Cholangiography was repeated six weeks after surgery. If no bile leakage or biliary stricture was observed, the biliary drains were removed.

In cases with AL, various treatment modalities e.g., conservative therapy with prolonged utilization of intraoperatively placed drains, computed tomography (CT)-guided drainages, percutaneous biliary drainages (PBD), relaparotomy, or a combination of these were chosen on a case-by-case basis. Treatment success was defined as complete closure of the leakage, while treatment failure is defined as associated mortality due to bile peritonitis, liver failure or vascular erosions.

Each patient was assessed for both tumor recurrence and signs of biliary stenosis regularly by the referring oncologist or the local outpatient clinic. If AS occurred during follow-up, various treatment modalities e.g., conservative therapy, PBD or relaparotomy were performed. Treatment success in stenosis is defined as a complete control of the clinical symptoms or complete resolution of the stenosis, while treatment failure is defined as associated mortality due to cholangitis or liver failure. Local tumor recurrence, as the underlying pathology of AS, was assessed and confirmed by imaging and elevated serum CA 19-9. Patients with 90-day mortality were excluded from the statistical analysis regarding AS.

### 2.3. Statistical Analysis

The statistical endpoints of this study were AL and AS in pCCA patients undergoing surgical resection. Data derived from continuous variables are presented as mean and standard deviation. Associations between perioperative variables and the statistical endpoints were assessed by means of univariate binary logistic regressions. For this purpose, nominal and categorical data were recoded into a scaled dummy variable. Median follow up was accessed with the reverse Kaplan–Meier method. The level of significance was set to *p* < 0.05 and *p*-values are given for two-sided testing. Analyses were performed using SPSS Statistics 24 (IBM Corp., Armonk, NY, USA).

## 3. Results

### 3.1. Preoperative, Operative and Postoperative Data

A total of 114 patients with a mean age of 65 ± 10 years and mean BMI of 26 ± 5 kg/m^2^ underwent curative surgery for pCCA at our institution from January 2011 to June 2019. More than half of the cohort (52.6%, 60/114) had a preoperative performance status ASA III or higher. The majority of the tumors were classified as Bismuth Type III or IV (89.5%; 102/114). Of all patients, 89.1% (23/88) had documented episodes of preoperative cholangitis. Portal vein reconstruction was carried out in all patients (100%; 114/114)), arterial resection in 9.6% (11/114) and a simultaneous pancreaticoduodenectomy in 7.9% (9/114) cases. None of the patients underwent laparoscopic liver resection and the mean operative time was 427 ± 94 min. The cohort had a mean hospital stay of 27 ± 20 days after surgery. The mean postoperative comprehensive complication index (CCI) was 45 ± 33 and the 90-day mortality 12.3% (14/114). Median follow-up was 2.3 years. Detailed clinical characteristics are outlined in Table 1.

AL was present in 11.4% (13/114) of the patients and was diagnosed 11 ± 7 days after surgery. A subset of three patients (23.0%, 3/13) died postoperatively due to the leakage, while the remaining 77.0% (10/13) were successfully treated. AS occurred in 11.0% (11/100) of the individuals eligible for follow-up and was diagnosed 2.4 ± 2.3 years after surgery. In 72.7% (8/11) of the cases with postoperative stenosis, local tumor recurrence was the underlying pathology, while the rest of these patients (27.3%, 3/11) had benign anastomotic strictures. A subgroup of two patients (18.2%, 2/11) deceased due to clinical consequences of the AS, while 81.8% (9/11) were successfully treated. Further, R1 resections were observed in 18.2% (2/11) of patients with AS and in 12.4% (11/89) of patients without AS (*p* = 0.558). Detailed data regarding AL or AS as well as the applied treatment modalities are presented in Table 2.

### 3.2. Univariate Analysis of Postoperative Leakage and Stenosis of Hepaticojejunostomies

A univariate binary logistic regression was carried out for AL including all available pre-, intra- and postoperative variables (Table 3). This analysis showed significant associations between the comprehensive complications index (CCI; Exp(B) = 1.02; *p* = 0.046), intensive care days (Exp(B) = 1.03; *p* = 0.048) and the duration of hospitalization (Exp(B) = 1.05; *p* = 0.001) and the presence of a AL. A similar analysis regarding AS was also carried out (Table 3). Here, the necessity of more than one HJ (HR = 5.35, *p* = 0.014) to reconstruct bile flow after liver resection was the single variable associated with AS in this analysis.

## 4. Discussion

Major liver resections with the concomitant resection of the extrahepatic bile duct and en-bloc lymphadenectomy have evolved as the gold standard of treatment for patients with pCCA [10,18]. Although, HJ is a major step of the complex surgical procedure, short- and long-term complications such as leakage and stenosis and their impact on clinical outcomes are yet to be reported. In fact, to the best of our knowledge, this is the first study analyzing the clinical course and risk factors for postoperative AL and AS after surgical resection for pCAA.

In a large monocentric European cohort, we observed AL in 11% as well as in 11% of our patients. While some statistical significances were detected in univariate analysis, none of the identified variables were meaningful enough or suitable for clinical decision-making. Treatment for both conditions was successful in most patients with treatment failures in 23% of the cases with AL and 18% of the cases with AS.

Bile leakage is considered a major postoperative complication after liver surgery leading to prolonged hospitalization und increased morbidity [21,22]. Some previous reports also associate bile leakage with postoperative mortality due to abdominal infection, sepsis and liver failure [23,24]. Incidence and risk factors of bile leakage in liver surgery have been extensively investigated [25,26,27]. Interestingly, cholangiocarcinoma as an entity and the necessity of a HJ during the procedure have already been identified as risk factors of postoperative bile leakage in various cohorts [21,28]. While previous literature does only comprise heterogenous cohorts with various tumor entities treated by a broad range of surgical procedures, our data represents the clinical outcomes of a large single-center cohort of pCCA patients. We report an overall incidence of bile leakage from the HJ in 11% of the cases which might appear excessive. This can be sufficiently explained by the complexity of the HJ with more than one bile duct ostia in 78% and more than three bile duct ostia in 22% of the cases and the need of extended liver resections in 81% of all patients. In addition, our figures are not comparable to the low incidence of bile leaks from the HJ after pancreaticoduodenectomy [11,12,13]. It should also be taken into account, that due to our standard approach with trans-anastomotic drainages and subsequent cholangiography, we are able to detect minor leaks without major clinical consequences. In fact, in nearly half of the particular cases (46.2%, 6/13), the AL was successfully managed in a conservative way by leaving the intra-abdominal drains in situ for a prolonged period. 

Our analysis showed CCI, ICU time and hospitalization to be significantly associated with the occurrence of AL. However, all significant variables are postoperative measures of complications and are rather a consequence of the leakage than explanatory predictors. Interestingly, no other preoperative or intraoperative variable reflecting technical difficulty of the anastomosis achieved statistical significance in our analysis. This finding has special implications for the surgical technique in pCCA. Left-sided hepatectomies are considered to be more prone for biliary leaks of the HJ due to likelihood of more separate bile duct ostia compared to right-sided hepatectomies [4,18,29]. In a recent report of our group, we were able to demonstrate that oncological outcome after left-sided hepatectomy is similar to right-sided hepatectomy for pCCA [18]. In this analysis with respect to AL rates, the results after right- vs. left-sided hepatectomies were comparable. Since the more complex biliary reconstruction used to be an argument favoring right-sided hepatectomy over left-sided hepatectomy, this finding is of upmost importance for preoperative planning of the surgical procedure as in a subset of patients presenting with pCCA both left-sided and right-sided hepatectomy are technically feasible (Figure 1).

The available literature describing biliary strictures after surgery is also sparse and contradicting. A large multicentric analysis of the North-American Medicare data using a heterogeneous cohort of patients who underwent surgery requiring a biliary-enteric anastomosis observed a cumulative incidence of stricture of 12.5% at 2 years [15]. In contrast, a large single-institution analysis from the Johns Hopkins Medical Institutions examined the incidence of biliary strictures after pancreaticoduodenectomy for benign and malignant periampullary disease and detected biliary strictures in only 2.6% of the patients [16]. While the above-mentioned multicenter analysis did not differentiate between benign and malign strictures, the latter study of House et al. found local tumor recurrence to be the underlying pathology in less than 10% of the cases with stricture. Our rate of AS in 11% of the overall cohort in this study might appear high, however, the vast majority (73%) of patients presenting with AS were later diagnosed with local tumor recurrence. Benign strictures were only present in 3% of the patients which is comparable to the report of House et al. [16]. The only significant parameter in our univariate analyses of risk factors was the necessity of more than one HJs. Nevertheless, we consider this founding doubtful or a random effect since most of our AS were actually caused by tumor recurrence and not by benign strictures and are therefore not eligible for an analysis reflecting the technical aspect of the surgical procedure.

Importantly, the majority of patients with AS were successfully treated with PBD. This is a clinically particularly meaningful information, as surgical therapy is technically difficult in the situation of local recurrence. As the local recurrence arises in close proximity to the portal vein and hepatic artery the tumor is usually technically not resectable especially in patients who underwent extended liver resections or trisectionectomy during the initial surgical therapy. Another challenging issue in AS to our experience is the differentiation between benign strictures and local recurrence in the early disease course with no detectable tumor mass. Due to the usually unfavorable location of the local recurrence, a small tumor mass can already result in stenosis of the hepaticojejunostomy leading to an often very similar clinical presentation of local recurrence and benign stenosis. In our experience, highly elevated CA19-9 can be an early sign of a malign etiology of the AS. Further, the treatment of the AS is becoming even more complex with the increasing involvement of the bile ducts as the tumor progresses and patients with local recurrence develop a detectable tumor mass over time. As the majority of AS was based on local recurrence in our cohort, we therefore recommend high quality cross-sectional imaging in every case of AS.

The overall 90-day mortality of the cohort was 12.3% which might appear high but is in line with the findings from previous reports [6,7]. It must be emphasized, that our cohort represents a high amount of patients with significant comorbidities (50% ASA III or higher) and extensive tumor burden (90% Bismuth Type III tumor or higher). In addition, trisectionectomy was the operative procedure in 30% of all cases and portal vein reconstruction was carried out in all patients as well as arterial resection and concomitant pancreaticoduodenectomy were likewise necessary in a significant number of our patients.

Like with all perioperative outcome studies, our analysis has potential limitations. All patients included in this study were treated at a single institution reflecting our distinct surgical technique which might not be comparable to other centers. This particularly accounts for our strategy to conduct portal vein resection and reconstruction in all cases. In addition, the study is based on retrospective data that was not obtained during a controlled prospective clinical trial. Furthermore, our sample size is relatively small and the number of events (leakage and stenosis) is, therefore, limited. However, due to the technical varieties in HJ technique among different centers and diverse clinical standards regarding trans-anastomotic and abdominal drains, a multi-center analysis with a higher sample size would also be biased in terms of the largely different surgical techniques. For this reason, we consider a homogenous surgical approach within our cohort as a major strength of the analysis.

Notwithstanding the aforementioned limitations, we have identified AL and AS of the HJ as a frequent and important complication in patients undergoing liver resection for pCCA. Technical difficulty of the HJ showed no association with the occurrence of either of these complications and both, AL and AS, can be treated by conservative, interventional or surgical therapy with a high success rate.

## Figures and Tables

**Table 1:** Clinical characteristics (*n* = 114).

Demographics	Mean ± SD
Gender, m/f (%)	76 (66.7)/38 (33.3)
Age (years)	65 ± 10
BMI (kg/m^2^)	26 ± 5
Portal vein embolization, *n* (%)	46 (39.7)
ASA, *n* (%)	
I	6 (5.3)
II	48 (42.1)
III	57 (50.0)
IV	3 (2.6)
V	0
Bismuth Type, *n* (%)	
I	3 (2.6)
II	9 (7.9)
IIIa	32 (28.1)
IIIb	32 (28.1)
IV	38 (33.3)
EBD, *n* (%)	
None	24 (21.1)
Unilateral	66 (57.9)
Bilateral	24 (21.1)
PBD, *n* (%)	
None	86 (75.4)
Unilateral	22 (19.3)
Bilateral	6 (5.3)
Preoperative cholangitis	
Yes	32 (28.8)
No	79 (71.2)
sFLR (%)	54 ± 20
**Preoperative liver function**	
Albumin (g/dL)	38 ± 7
AST (U/L)	103 ± 289
ALT (U/L)	124 ± 209
GGT (U/L)	573 ± 526
Total bilirubin (mg/dL)	2.1 ± 2.6
Platelet count (1/nL)	324 ± 139
Alkaline Phosphatase (U/L)	311 ± 233
Prothrombine time (%)	93 ± 17
INR	1.06 ± 0.18
Hemoglobin (g/dL)	12.3 ± 1.6
CRP (mg/dL)	30 ± 39
**General operative data**	
Laparoscopic resection, *n* (%)	0
Operative time (minutes)	427 ± 94
Operative procedure, *n* (%)	
Left hepatectomy	11 (9.6)
Extended left hepatectomy	35 (30.7)
Left trisectionectomy	7 (6.1)
Right hepatectomy	11 (9.6)
Extended right hepatectomy	21 (18.4)
Right trisectionectomy	29 (25.4)
Concomitant pancreatic resection, *n* (%)	9 (7.9)
Portal vein reconstruction, *n* (%)	114 (100)
Arterial resection, *n* (%)	11 (9.6)
Intraoperative blood transfusion (units)	1 ± 2
Intraoperative FFP (units)	3 ± 3
**Hepaticojejunostomy characteristics**	
Number of bile duct ostia, *n* (%)	
1	25 (21.9)
2	39 (34.2)
3	25 (21.9)
≥4	25 (21.9)
Number of hepaticojejunostomies, *n* (%)	
1	93 (81.6)
≥2	21 (18.4)
Number of bile duct drains, *n* (%)	
0	10 (8.8)
1	50 (43.9)
2	50 (43.9)
≥3	4 (3.5)
Postoperative anastomotic leakage, *n* (%)	13 (11.4)
Postoperative anastomotic stenosis *, *n* (%)	11 (11.0)
Cause of postoperative stenosis, *n* (%)	
Benign	3 (27.3)
Malign	8 (72.7)
**Pathological data**	
R category, *n* (%)	
R0	97 (85.1)
R1	17 (14.9)
T category, *n* (%)	
I/II	76 (67.3)
III/IV	37 (32.7)
N category, *n* (%)	
N0	67 (59.3)
N1	46 (40.7)
**Postoperative Data**	
Intensive care, days	6 ± 15
Hospitalization, days	27 ± 20
Postoperative complications, *n* (%)	
Clavien–Dindo ≤ IIIa	65 (57.0)
Clavien–Dindo ≥ IIIb	49 (43.0)
CCI	45 ± 33
90-day mortality, *n* (%)	14 (12.3)

Data presented as mean and standard deviation if not noted otherwise.; ALT, alanine aminotransferase; ASA, American society of anesthesiologists classification; AST, aspartate aminotransferase; BMI, body mass index; CCI, comprehensive complication index; CRP, c-reactive protein; EBD, endoscopic biliary drainage; FFP, fresh frozen plasma; GGT, gamma glutamyltransferase; INR, international normalized; PBD, percutaneous biliary drainage; ratio; PVE, portal vein. embolization. sFLR, standardized future liver remnant. * Only patients without 90-day mortality were included in the analysis.

**Table 2:** Specific treatment and treatment success in postoperative anastomotic leakage or stenosis of hepaticojejunostomies.

No	Leakage/Stenosis	Interval from Surgery to Event (Days/Months)	1st Treatment	2nd Treatment	3rd Treatment	Treatment Success (y/n)	Cause of Stenosis
1	Leakage	27	Conservative	n. a.	n. a.	Yes	n. a.
2	Leakage	22	Conservative	n. a.	n. a.	Yes	n. a.
3	Leakage	9	Conservative	n. a.	n. a.	Yes	n. a.
4	Leakage	6	Conservative	n. a.	n. a.	Yes	n. a.
5	Leakage	6	Conservative	n. a.	n. a.	Yes	n. a.
6	Leakage	5	Conservative	n. a.	n. a.	Yes	n. a.
7	Leakage	7	CT drainage	PBD	n. a.	Yes	n. a.
8	Leakage	11	Surgery	n. a.	n. a.	Yes	n. a.
9	Leakage	5	Surgery	n. a.	n. a.	Yes	n. a.
10	Leakage	4	Surgery	PBD	n. a.	Yes	n. a.
11	Leakage	18	Surgery	n. a.	n. a.	No	n. a.
12	Leakage	8	Surgery	n. a.	n. a.	No	n. a.
13	Leakage	14	Surgery	Surgery	n. a.	No	n. a.
1	Stenosis	83	PBD	n. a.	n. a.	Yes	Malign
2	Stenosis	77	PBD	n. a.	n. a.	Yes	Malign
3	Stenosis	43	PBD	n. a.	n. a.	Yes	Malign
4	Stenosis	5	PBD	n. a.	n. a.	Yes	Malign
5	Stenosis	24	PBD	Surgery	n. a.	Yes	Malign
6	Stenosis	11	PBD	PBD	PBD	Yes	Malign
7	Stenosis	9	PBD	n. a.	n. a.	No	Malign
8	Stenosis	8	Conservative *	n. a.	n. a.	No	Malign
9	Stenosis	18	PBD	PBD	n. a.	Yes	Benign
10	Stenosis	11	PBD	n. a.	n. a.	Yes	Benign
11	Stenosis	24	PBD	n. a.	n. a.	Yes	Benign

Specific treatment and treatment success are shown for each patient who presented with anastomotic leakage or anastomotic stenosis of the hepaticojejunostomy. Interval from surgery to event is shown in days for anastomotic leakage and in months for anastomotic stenosis. Treatment success in case of anastomotic leakage is defined as complete closure of the leakage, while treatment failure is defined as associated mortality. Treatment success in anastomotic stenosis is defined as a complete control of the clinical symptoms or complete resolution of the stenosis, while treatment failure is defined as associated mortality due to cholangitis or liver failure. * Interventional treatment was offered but refused by the patient. N.a., not applicable; PBD, percutaneous biliary drainage.

**Table 3:** Univariable binary logistic analysis of postoperative anastomotic leakage and stenosis of hepaticojejunostomies.

	Anastomotic Leakage		Anastomotic Stenosis	
	Exp (B)/HR	*p*-Value	Exp (B)/HR	*p*-Value
Sex (male = 1)	1.29	0.677	2.61	0.138
Age	1.02	0.517	1.04	0.252
BMI	0.95	0.427	1.02	0.781
ASA scale	0.73	0.490	2.60	0.086
Bismuth Type				
I	0.00	0.999	0.00	0.999
II	0.00	0.999	1.25	0.853
III	0.94	0.923	0.90	0.878
IV	1.00		1.00	
EBD				
None	1.00		1.00	
Unilateral	0.70	0.635	0.58	0.478
Bilateral	1.40	0.683	1.27	0.790
PBD				
None	1.00		1.00	
Unilateral	0.76	0.736	1.095	0.913
Bilateral	1.52	0.715	0.00	0.999
Preoperative cholangitis (no = 1)	0.41	0.267	2.79	0.118
sFLR	4.17	0.330	3.20	0.446
Albumin	1.00	0.963	0.96	0.491
AST	1.00	0.969	1.00	0.691
ALT	1.00	0.771	0.993	0.289
GGT	1.00	0.403	1.00	0.975
Bilirubin	1.13	0.151	1.04	0.721
Alkaline phosphatase	1.00	0.524	1.00	0.733
Platelet count	1.00	0.400	1.00	0.721
Prothrombin time	1.01	0.541	0.99	0.645
INR	4.66	0.225	1.92	0.653
Hemoglobin	0.82	0.278	0.73	0.461
Operative time	1.00	0.193	1.01	0.111
Blood transfusions (no = 1)	2.69	0.118	2.05	0.279
FFP (no = 1)	2.01	0.311	6.48	0.081
Surgical resection				
Right hepatectomy	0.00	0.999	0.50	0.548
Left hepatectomy	1.81	0.464	0.00	0.999
Extended right hepatectomy	0.51	0.436	0.25	0.219
Extended left hepatectomy	1.00		1.00	
Right trisectionectomy	0.36	0.232	0.48	0.336
Left trisectionectomy	0.00	0.207	0.00	0.999
Resection type				
Right-sided resection	1.00		1.00	
Left-sided resection	2.92	0.091	1.47	0.548
Pancreatic resection (no = 1)	0.000	0.999	0.00	0.999
Arterial resection (no = 1)	3.49	0.098	1.01	0.991
Number of bile duct ostia				
1	1.00		1.00	
2	1.31	0.763	3.29	0.302
3	2.19	0.393	1.21	0.895
≥4	1.57	0.639	6.05	0.114
Number of hepaticojeunostomy				
1	1.00		1.00	
≥2	1.38	0.647	5.35	0.014
Number of bile duct drains, *n* (%)				
0	0.00	0.999	1.83	0.618
1	1.00		1.00	
2	1.00	1.000	3.02	0.129
≥3	2.44	0.469	0.00	0.999
R category (R0 = 1)	1.86	0.386	1.58	0.591
T category (I/II = 1)	1.91	0.279	1.22	0.761
N category (N0 = 1)	1.53	0.491	0.132	0.070
Clavien Dindo ≥ IIIb (≤ IIIa = 1)	2.34	0.160	0.67	0.571
CCI	1.02	0.046	0.99	0.508
90-day mortality (no = 1)	0.56	0.597	n. a.	n. a.
Intensive care days	1.03	0.048	0.98	0.628
Hospitalization	1.05	0.001	1.00	0.907

Various parameters are tested for association with leakage or stenosis of the hepaticojejunostomies. The overall cohort was analyzed for leakage of the hepaticojejunostomy, while only patients without 90-day mortality were analyzed for stenosis of the hepaticojejunostomy. ALT, alanine aminotransferase; ASA, American society of anesthesiologists classification; AST, aspartate aminotransferase; BMI, body mass index; CCI, comprehensive complication index; EBD, endoscopic biliary drainage; FFP, fresh frozen plasma; GGT, gamma glutamyltransferase; PBD, percutaneous biliary drainage; HR, hazard ratio; INR, international normalized ratio; sFLR, standardized future liver remnant.

## Abbreviations

AL: Anastomotic leakage
ALT: Alanine aminotransferase
AS: Anastomotic stenosis
AP: Alkaline phosphatase
ASA: American society of anesthesiologists
AST: Aspartate aminotransferase
BMI: Body mass index
CCI: Comprehensive complication index
CRP: C-reactive protein
CT: Computed tomography
CUSA: Cavitron Ultrasonic Surgical Aspirator
EBP: Endoscopic biliary drainage
ERCP: Endoscopic retrograde cholangiopancreatography
FFP: Fresh frozen plasma
FLR: Future liver remnant
GGT: Gamma glutamyltransferase
HJ: Hepaticojejunostomy
INR: International normalized ratio
LiMAx: Maximum liver function capacity
MRCP: Magnetic resonance cholangiopancreatography
MRI: Magnetic resonance imaging
NPV: Negative predictive value
PBD: Percutaneous biliary drainage
pCCA: Perihilar cholangiocarcinoma
PVE: Portal vein embolization
RWTH: Rheinisch-Westfälische Technische Hochschule
sFLR: Standardized future liver remnant
